# Peking prognostic score is a useful prognostic factor in patients with gastric cancer liver metastases receiving hepatectomy

**DOI:** 10.3389/fnut.2022.976364

**Published:** 2022-09-30

**Authors:** Jianping Xiong, Yunzi Wu, Haitao Hu, Wenzhe Kang, Yang Li, Peng Jin, Xinxin Shao, Weikun Li, Yibin Xie, Yantao Tian

**Affiliations:** Department of Pancreatic and Gastric Surgery, National Cancer Center/National Clinical Research Center for Cancer/Cancer Hospital, Chinese Academy of Medical Sciences and Peking Union Medical College, Beijing, China

**Keywords:** gastric cancer, liver metastases, hepatectomy, Peking prognostic score, prognostic scoring system

## Abstract

**Background:**

The present work evaluated how Peking prognostic score (PPS), the new prognostic index determined according to sarcopenia and lymphocyte-to-C-reactive protein ratio (LCR), was a prognostic factor for patients with gastric cancer liver metastases (GCLM) who received hepatectomy.

**Methods:**

This work extracted information about patients with GCLM who underwent hepatectomy from June 2012 to May 2018. The PPS of the patients was calculated from sarcopenia status and LCR before surgery, and patients were then divided into three groups based on their PPS. This work also carried out univariate and multivariate analyses for identifying variables that were linked with overall survival (OS) together with recurrence-free survival (RFS) after hepatectomy among three groups according to PPS.

**Results:**

This work included 108 GCLM cases who received hepatectomy. All cases were classified into 3 groups, i.e., 26 (24.1%), 48 (44.4%), and 34 (31.5%) in groups 0–2, separately. PPS exhibited positive relation with age (*p* < 0.001), body mass index (BMI; *p* = 0.012), and liver metastasis number. The relapse rate after hepatectomy in patients with GCLM was 69.4%. Additionally, the remnant liver relapse rates of groups 0–2 were 80.0, 68.7, and 53.5%. Patients in group 0 had significantly increased remnant liver relapse rates when compared with those in groups 0 and 1. PPS was significantly related to relapse patterns (*p* = 0.003). Relative to group 0, those of the other 2 groups showed dismal OS [hazard ratio (HR) = 3.98, 7.49 for groups 1 and 2; *p* < 0.001] along with RFS (HR = 3.65, 5.33 for groups 1 and 2; *p* < 0.001). As revealed by multivariate analysis, PPS independently predicted OS (*p* < 0.001) together with RFS (*p* < 0.001).

**Conclusion:**

The PPS could be an easy nutrition-inflammation prognostic scoring system and an independent preoperative predictor of survival for GCLM cases after hepatectomy.

## Introduction

Gastric cancer (GC) accounts for the 5th cancer in terms of its incidence, which affects 1,033,701 people every year. Additionally, GC accounts for the 3rd place among the frequent factors leading to cancer-related death, and 782,685 death cases are reported annually (1). Despite the numerous efforts conducted on GC diagnosis and treatment, its survival decreases due to distant metastases (DM). The liver represents a frequent organ subject to DM of GC, with the GC liver metastasis (GCLM) rate of 9.9–18.7% (2, 3). For GCLM cases, they have a median survival of approximately 7–12 months (4). Long-term survival can hardly be achieved among GCLM cases, although first-line chemotherapy is applied (4). As palliative chemotherapy has poor overall survival (OS) outcomes, hepatectomy is widely analyzed to be the appropriate way for improving patient survival. Numerous articles suggest that hepatectomy is advantageous for GCLM in the last 20 years (5–7). Some recent large articles suggest that for GCLM receiving hepatectomy, the 5-year OS was 31.1–39.5%(8, 9). However, hepatectomy is associated with a high relapse rate; therefore, determining hepatectomy timing and indications for GCLM cases is not easy. Consequently, it is essential to develop approaches to assess the hepatectomy feasibility in GCLM cases.

In recent years, inflammatory, immune, and nutritional statuses, which are the host-associated factors, receive wide attention in the prediction of postoperative outcomes within diverse cancer types. Typically, platelet-to-lymphocyte ratio (PLR), lymphocyte-to-monocyte ratio (LMR), and C-reactive protein ratio-albumin ratio (CAR) along with neutrophil-to-lymphocyte ratio (NLR) have been identified as factors predicting the prognosis of cancers. As reported recently, low lymphocyte-to-C-reactive protein ratio (LCR) independently predicted OS and recurrence-free survival (RFS) among GC cases who underwent gastrectomy (10, 11). In addition, LCR showed the greatest sensitivity to predict survival when compared with other inflammation-related scores (11). Sarcopenia, one of the age-related muscle mass, strength, and functional losses, is becoming a severe medical problem among the aging societies (12). It can be usually detected in GC cases, and its incidence is more than 6.8–57.7% among patients with GC (13). Additionally, it shows significant relation with the poor long-term prognosis among patients with GC receiving surgery (14). Our previous work indicated that sarcopenia effectively predicted prognosis of recurrence in patients with GCLM receiving hepatectomy (15). The Peking prognostic score (PPS), first proposed by Xiong et al., consists of sarcopenia and LCR and shows high relation to GC prognosis (16). Additionally, it exhibits increased accuracy when compared with additional prognostic factors for the prediction of survival (16). Nonetheless, the association of PPS with long-run patients with GCLM survival after hepatectomy for GCLM is still unclear.

This article assessed whether PPS preoperatively affected the long-run survival of GCLM after hepatectomy.

## Materials and methods

This article obtained approval from the Ethics Committee of the National Cancer Center/National Clinical Research Center for Cancer/Cancer Hospital, Chinese Academy of Medical Sciences, and Peking Union Medical College (NCC2020C-220). Each experiment was carried out following Transparent Reporting of a Multivariable Prediction Model for Individual Prognosis or Diagnosis and Declaration of Helsinki.

### Research design and objects

**FIGURE 1:**
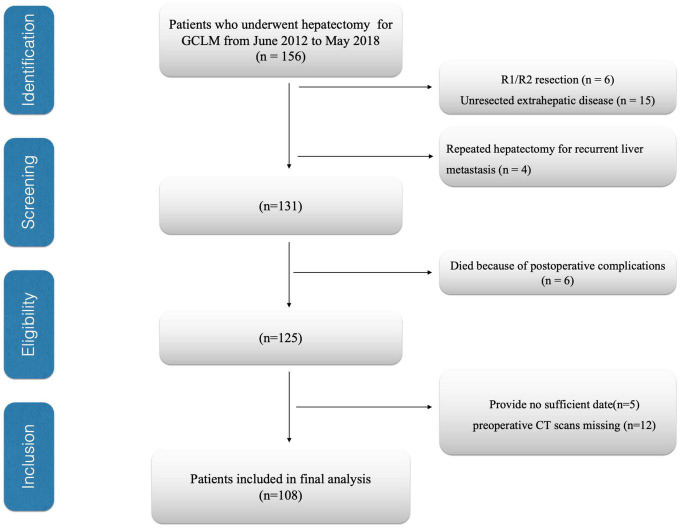
Flow diagram of patients. GCLM, gastric cancer liver metastasis; CT, computed tomography.

This study assessed patients with GCLM who underwent hepatectomy in the National Cancer Center/Cancer Hospital, Chinese Academy of Medical Sciences and Peking Union Medical College during June 2012–May 2018. This work set patient exclusion criteria below (1) those with an unresectable extrahepatic lesion (*n* = 15), (2) those who received several hepatectomies due to liver metastatic relapse (*n* = 4), (3) patients who underwent R1/R2 resection (*n* = 6), (4) patients having inadequate or inaccurate medical records (*n* = 5), (5) preoperative computed tomography (CT) scans missing (*n* = 12), and (6) those who were dying due to complications after surgery (*n* = 6). Finally, 108 cases were enrolled in the cohort (Figure 1). In this study, resected liver segment number ≥ 3 was deemed as major hepatectomy, while that < 3 as a minor hepatectomy (17). Recurrences were categorized as remnant liver and extrahepatic sites. Extrahepatic site recurrence included local (primary site), lymph node, lung, bone/brain, and peritoneal dissemination recurrence. Follow-up after surgery was carried out every 3 months within the first 2 years, whereas every half a year thereafter. This study deemed the primary end point OS as the period between surgery and all-cause mortality or final follow-up, whereas the secondary end point RFS was the period between surgery and mortality or disease relapse.

### Peking prognostic score and other prognostic scoring systems

Muscle quality was assessed ahead of time for identifying sarcopenia cases. CT was also employed for the accurate assessment of muscle mass. Additionally, the CT-based sarcopenia thresholds for gender-specific skeletal muscle index (SMI) of the third lumbar vertebra (L3) were ≤ 40.8, 34.9 cm^2^/m^2^ for men and women, separately, as proposed by Zhuang for Chinese GC population (18). The PPS model consisted of two parameters, namely, LCR and sarcopenia status. According to Xiong et al’s method, the scores were 0, 1, 2, and 3 for cases with LCR > 6,000 without sarcopenia, LCR > 6,000 with sarcopenia, LCR ≤ 6,000 without sarcopenia, and LCR ≤ 6,000 with sarcopenia, separately. All cases were classified into three groups based on their PPS scores: cases with the scores of 0, 1/2, and 3 were classified as groups 0–2, separately (Table 1).

**TABLE 1:** The definition of the Peking prognostic score.

Scoring system	Score	Group
**The PPS**		
Sarcopenia (No) and LCR (> 6,000)	0	0
Sarcopenia (Yes) and LCR (> 6,000)	1	1
Sarcopenia (No) and LCR (≤ 6,000)	2	1
Sarcopenia (Yes) and LCR (≤ 6,000)	3	2

LCR, lymphocyte-to-C-reactive protein ratio.

### Statistical methods

Kaplan–Meier (KM) survival analysis was conducted, whereas the log-rank test was employed in the analysis. This work also conducted univariate and multivariate analyses by Cox proportional hazards regression model. All of those prognostic factors identified by univariate analysis upon *p* < 0.1 were incorporated into multivariate regression. *p* < 0.05 represented statistical significance. R software version 4.0.2 (R Foundation for Statistical Computing, Vienna, Austria) together with JMP statistical software package (SAS Institute, Inc., Cary, NC, United States) was adopted in statistical analysis.

## Results

### Patient features

This work included altogether 108 GCLM cases who received hepatectomy, among them, 84 (77.6%) were men and 24 (22.4%) were women (Table 2). The age at hepatectomy ranged from 57.9 to 73.6 (mean, 63.5) years. With regard to liver metastasis, 41 cases (38.0%) developed synchronous metastatic lesions, whereas 67 cases (62.0%) developed metachronous metastatic lesions (Table 2). In total, 72 cases (66.7%) developed solitary liver metastatic lesions, whereas 36 (33.3%) developed multiple liver metastatic lesions (Table 2). In total, 60 cases (55.7%) underwent neoadjuvant chemotherapy (NACT), whereas 73 (67.5%) underwent adjuvant chemotherapy (ACT) (Table 2). Patients were divided into 3 groups, i.e., 26 (24.1%), 48 (44.4%), and 34 (31.5%) in groups 0 (PPS = 0), 1 (PPS = 1/2), and 2 (PPS = 3), separately (Table 2). With regard to OS rates, they were 78.6, 45.8, and 33.4%, separately in GCLM cases who received hepatectomy, while their RFS rates at 1, 3, and 5 years were 51.7, 36.5, and 27.3%, separately.

**TABLE 2:** Association of PPS and clinicopathological characteristics in patients with GCLM after hepatectomy.

Clinicopathological features	All cases (*n* = 108)	Group 0 (*n* = 26)	Group 1 (*n* = 48)	Group 2 (*n* = 34)	*P*-value
Age ≥ 65.0 < 65.0	63 (58.7) 45 (41.3)	7 (26.9) 19 (73.1)	28 (58.3) 20 (41.7)	28 (82.3) 6 (17.7)	*P* < 0.001
Gender Male Female	84 (77.6) 24 (22.4)	19 (73.1) 7 (26.9)	38 (79.1) 10 (20.9)	27 (79.4) 7 (20.6)	0.769
BMI (kg/m2) < 18.5 ≥ 18.5	16 (14.8) 92 (85.2)	2 (7.7) 24 (92.3)	6 (12.5) 42 (87.5)	8 (23.5) 26 (76.5)	0.012
Serum albumin (g/dL) ≥ 3.5 < 3.5	83 (76.8) 25 (23.2)	42 (72.4) 5 (19.2)	42 (72.4) 11 (22.9)	44 (78.6) 9 (26.5)	0.091
CEA (ng/mL) ≥ 5.0 < 5.0	61 (56.5) 47 (43.5)	15 (57.6) 11 (42.4)	26 (54.2) 22 (45.8)	20 (58.8) 14 (51.2)	0.540
Tumor location of GC Upper Middle/Lower	34 (31.4) 74 (68.6)	9 (34.6) 17 (65.4)	14 (29.2) 34 (70.8)	11 (32.3) 23 (67.7)	0.601
Lauren Classification Intestinal-type Diffused-type Mixed	54 (50.0) 33 (30.5) 21 (19.5)	12 (46.1) 9 (34.6) 5 (19.3)	25 (52.0) 14 (29.2) 9 (18.8)	17 (50.0) 10 (29.4) 7 (20.6)	0.553
Timing of liver metastases metachronous synchronous	67 (62.0) 41 (38.0)	17 (65.3) 9 (34.7)	29 (60.4) 19 (39.6)	21 (61.7) 13 (38.3)	0.308
Number of liver metastases ≤ 1 >1	72 (66.7) 36 (33.3)	14 (53.8) 12 (46.2)	31 (64.6) 17 (35.4)	27 (79.4) 7 (20.6)	0.002
Maximum tumor size of the liver metastasis < 3 ≥ 3	68 (62.9) 40 (37.1)	17 (65.3) 9 (34.7)	28 (58.3) 20 (41.7)	24 (70.6) 10 (29.4)	0.304
Type of hepatectomy Minor Major	86 (79.6) 22 (20.4)	20 (80.8) 6 (19.2)	40 (83.3) 8 (16.7)	25 (76.4) 9 (23.6)	0.513
Neoadjuvant chemotherapy No Yes	48 (44.3) 60 (55.7)	12 (42.3) 15 (57.7)	20 (41.7) 28 (58.3)	17 (50.0) 17 (50.0)	0.446
Adjuvant chemotherapy No Yes (Systemic chemotherapy/HAIC/Systemic chemotherapy + HAIC)	35 (32.5) 73 (67.5)	9 (30.8) 18 (69.2)	18 (37.5) 30 (62.5)	10 (29.5) 24 (70.5)	0.217

PPS, Peking prognostic score; GCLM, gastric cancer liver metastasis; BMI, body mass index; GC, gastric cancer; HAIC, hepatic artery infusion chemotherapy.

### Associations of Peking prognostic score with clinicopathological characteristics

Table 2 presents the relation between PPS and patient clinicopathological characteristics. PPS was significantly related to advanced age (≥ 65.0 years; *p* < 0.001) together with decreased body mass index (BMI; < 18.5 kg/m^2^; *p* = 0.012). Additionally, PPS showed a significant relationship with liver metastasis number (*p* = 0.002). The relapse rate after hepatectomy in patients with GCLM was 69.4%. Additionally, the remnant liver relapse rates of groups 0–2 were 80.0, 68.7, and 53.5%. Patients in group 0 had significantly increased remnant liver relapse rates when compared with those in groups 0 and 1. PPS was significantly related to relapse patterns (*p* = 0.003; Table 3).

**TABLE 3:** Impact of PPS on the recurrence in patients with GCLM receiving hepatectomy.

	All cases (*n* = 75)	Group 0 (*n* = 15)	Group 1 (*n* = 32)	Group 2 (*n* = 28)	*P*-value
Patterns of recurrence Remnant liver Extrahepatic sites (Lung\Lymph node\Peritoneal dissemination\Local (primary site) \Bone/brain)	49 (65.3) 26 (34.7)	12 (80.0) 3 (20.0)	22 (68.7) 10 (31.3)	15 (53.5) 13 (46.5)	*P* = 0.003

PPS, Peking prognostic score; GCLM, Gastric Cancer Liver Metastasis.

### Clinical role of preoperative Peking prognostic score in patients with gastric cancer liver metastases

**FIGURE 2:**
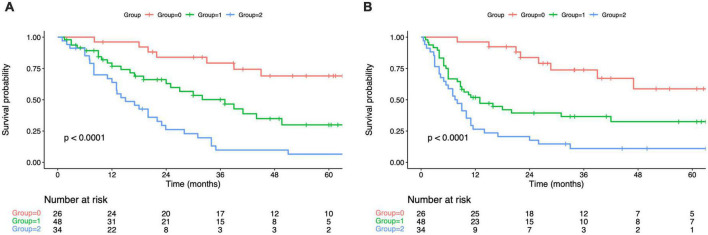
Kaplan–Maier curves of overall survival for each PPS group **(A)**. Kaplan–Maier curves of overall survival for each PPS group **(B)**. PPS, Peking prognostic score.

As revealed by KM survival analysis, OS and RFS were evidently reduced as the PPS group increased step-wise (OS, RFS: log-rank test, *p* < 0.001; Figures 2A,B). In comparison with group 0, those of groups 1/2 showed decreased OS and RFS. Upon multivariate regression, PPS independently predicted OS (HR = 2.12, 95% CI = 1.14–4.25, *p* < 0.001; HR = 4.76, 95% CI = 1.49–6.90, *p* < 0.001 for PPS groups 1 and 2 separately) and RFS (HR = 3.08, 95% CI = 1.29–4.38, *p* < 0.001; HR = 4.32, 95% CI = 1.75–6.87, *p* < 0.001 for PPS groups 1 and 2 separately; Tables 4, 5).

**TABLE 4:** Univariate and multivariate analysis of clinicopathologic variables in relation to overall survival in patients with GCLM after hepatectomy.

Clinicopathological features	Univariate analysis	*P*-value	Multivariate analysis	*P*-value
Age < 65.0 ≥ 65.0	Reference 1.42 (0.84, 3.76)	0.311		
Gender Male Female	Reference 0.85 (0.69, 3.19)	0.298		
BMI (kg/m2) ≥ 18.5 < 18.5	Reference 3.13 (1.47, 4.22)	< 0.001	Reference 1.90 (0.87, 2.83)	0.182
Serum albumin (g/dL) ≥ 3.5 < 3.5	Reference 2.15 (1.30, 3.96)	< 0.001	Reference 1.69 (1.24, 2.76)	0.002
CEA (ng/mL) ≥ 5.0 < 5.0	Reference 1.97 (1.24, 3.87)	< 0.001	Reference 1.41 (1.17, 2.63)	0.005
Tumor location of GC Upper Middle/Lower	Reference 0.85 (0.53, 4.10)	0.324		
Lauren Classification Intestinal-type Diffused-type Mixed	Reference 2.79 (0.74, 4.16) 1.92 (0.83, 4.63)	0.411 0.308		
Timing of liver metastases metachronous synchronous	Reference 1.61 (0.70, 3.48)	0.335		
Number of liver metastases ≤ 1 >1	Reference 2.25 (1.22, 3.76)	< 0.001	Reference 1.75 (1.29, 2.93)	< 0.001
Maximum diameter of the liver metastasis < 3 ≥ 3	Reference 2.74 (1.31, 4.02)	< 0.001	Reference 1.89 (1.13, 3.28)	0.008
Type of hepatectomy Minor Major	Reference 1.46 (0.82, 1.95)	0.420		
Neoadjuvant chemotherapy No Yes	Reference 0.83 (0.60, 1.84)	0.186		
Adjuvant chemotherapy No Yes (Systemic chemotherapy/HAIC/Systemic chemotherapy + HAIC)	Reference 0.72 (0.59, 0.96)	0.030	Reference 0.69 (0.52, 1.20)	0.101
PPS 0 1 2	Reference 3.98 (1.80, 8.02) 7.49 (2.23, 11.62)	< 0.001 < 0.001	Reference 2.12 (1.14, 4.25) 4.76 (1.49, 6.90)	< 0.001 < 0.001

PPS, Peking prognostic score; GC, gastric cancer; GCLM, gastric cancer liver metastasis; BMI, body mass index; HAIC, hepatic artery infusion chemotherapy.

**TABLE 5:** Univariate and multivariate analyses of clinicopathologic variables in relation to recurrence-free survival in patients with GCLM after hepatectomy.

Clinicopathological features	Univariate analysis	*P*-value	Multivariate analysis	*P*-value
Age < 65.0 ≥ 65.0	Reference 1.75 (0.79, 3.81)	0.462		
Gender Male Female	Reference 0.80 (0.53, 3.11)	0.337		
BMI (kg/m2) ≥ 18.5 < 18.5	Reference 3.62 (1.60, 4.78)	< 0.001	Reference 1.87 (0.75, 2.66)	0.340
Serum albumin (g/dL) ≥ 3.5 < 3.5	Reference 2.89 (1.65, 4.52)	< 0.001	Reference 1.90 (0.87, 3.01)	0.214
CEA (ng/mL) ≥ 5.0 < 5.0	Reference 2.14 (1.30, 3.95)	< 0.001	Reference 1.58 (1.23, 2.80)	0.002
Tumor location of GC Upper Middle/Lower	Reference 0.69 (0.58, 4.17)	0.411		
Lauren Classification Intestinal-type Diffused-type Mixed	Reference 3.11 (0.69, 4.01) 1.87 (0.72, 5.60)	0.409 0.515		
Timing of liver metastases metachronous synchronous	Reference 1.55 (0.78, 4.12)	0.442		
Number of liver metastases ≤ 1 >1	Reference 2.10 (1.28, 4.39)	< 0.001	Reference 1.82 (1.16, 2.70)	< 0.001
Maximum diameter of the liver metastasis < 3 ≥ 3	Reference 2.36 (1.36, 4.17)	< 0.001	Reference 1.43 (0.88, 3.05)	0.274
Type of hepatectomy Minor Major	Reference 1.67 (0.70, 2.13)	0.558		
Neoadjuvant chemotherapy No Yes	Reference 0.75 (0.69, 1.74)	0.116		
Adjuvant chemotherapy No Yes (Systemic chemotherapy/HAIC/Systemic chemotherapy + HAIC)	Reference 0.78 (0.60, 1.96)	0.330		
PPS 0 1 2	Reference 3.65 (1.80, 7.67) 5.33 (2.10, 12.79)	< 0.001 < 0.001	Reference 3.08 (1.29, 4.38) 4.32 (1.75, 6.87)	< 0.001 < 0.001

PPS, Peking prognostic score; GC, gastric cancer; GCLM, gastric cancer liver metastasis; BMI, body mass index; HAIC, hepatic artery infusion chemotherapy.

Factors significantly related to OS upon univariate analysis were BMI (*p* < 0.001), serum albumin (*p* < 0.001), carcinoembryonic antigen (CEA) levels (*p* < 0.001), ACT (*p* = 0.030), liver metastasis number (*p* < 0.001), and maximal liver metastasis size (*p* < 0.001; Table 4). Multivariate analysis identified serum albumin (HR = 1.69; 95% CI = 1.24–2.76; *p* = 0.002), liver metastasis number (HR = 1.75; 95% CI = 1.29–2.93; *p* < 0.01), and maximal liver metastasis size (HR = 1.89; 95% CI = 1.13–3.28; *p* = 0.008) to be independent factors predicting OS (Table 4).

Factors significantly related to RFS upon univariate analysis were serum albumin (*p* < 0.001), liver metastasis number (*p* < 0.001), and maximal liver metastasis diameter (*p* = 0.002; Table 5). Multivariate analysis identified liver metastasis number (HR = 1.82; 95% CI = 1.16–2.70; *p* < 0.001) and maximal liver metastasis size (HR = 1.43; 95% CI = 1.08–3.05, *p* = 0.004) to be independent factors predicting RFS (Table 5).

## Discussion

This article analyzed whether PPS was of prognostic value in GCLM cases receiving hepatectomy and identified PPS as the factor independently predicting long-term survival of GCLM cases. According to this work, PPS was potently linked to OS and RFS, typically, patients who had increased PPS values had reduced OS and RFS. PPS was linked to old age (≥ 65.0 years) and declined BMI (< 18.5 kg/m^2^) together with liver metastasis number (> 1).

Liver metastasis may take place within around 5–14% of cases receiving GC surgery (2). Liver metastasis treatment plays a critical role in improving the prognosis of GC cases. GCLM cases can be traditionally treated by palliative chemotherapy (19). However, numerous articles suggest that liver metastatic resection can significantly improve some patient survival in the last several years. As reported in Far Eastern Research, GCLM survival was posthepatectomy increased when compared with Western studies (2). According to the Chinese consensus on the diagnosis and treatment of GC with liver metastases, type I patients can choose surgical treatments (3). The criteria are as follows: (1) gastric tumors: depth of invasion ≤ T4a; lymph node metastases within D2 lymph node dissection (not including Bulky N2). (2) Bulky N2: at least one node of ≥ 3 cm in diameter or at least three consecutive nodes each of diameter ≥ 1.5 cm, along the coeliac, splenic, common, or proper hepatic arteries. Liver metastases: 1–3; maximal diameter ≤ 4 cm or limited to one liver lobe without involving important vessels or bile ducts. (3) Assessment of resectability: technological resectability of liver metastases judged by a hepatobiliary surgeon; meets the resection standard of hepatic reservation function assessment (3). Nevertheless, 5-year OS following hepatectomy was 27.7–42.3%, while the relapse rates after hepatectomy were 60–75% (9, 20, 21). Consequently, predicting and assessing relapse has been considered among GCLM cases receiving hepatectomy.

According to the results of recent studies, many favorable prognostic factors have been suggested, such as primary cancer (lower T and N stage, no lympho-vascular or serosal invasion), burden of hepatic disease (≤ 3 metastases, unilobar involvement, greatest lesion < 5 cm, and negative resection margins), and lower CEA and CA19.9 levels (9, 20, 21). An increasing number of articles have analyzed the relationship between malnutrition or systemic inflammation and tumor genesis, progression, and metastasis, which has been verified in different cancers that include GC, resulting in looking for markers related to nutrition and inflammation and developing the new prognosis scoring system. Serum CRP has been the typical marker reflecting systemic inflammatory responses, in addition, an increased CRP level related to poor prognosis for GC. According to Okugawa and Nakamura, LCR served as the prognostic biomarker for GC (10, 11), resectable colorectal cancer (CRC) (22), and unresectable metastatic CRC (23). Moreover, LCR is recently suggested to show the highest accuracy in predicting survival when compared with inflammatory scores, such as NLR, CAR, LMR, or PLR (11, 22, 23). Sarcopenia, the skeletal muscle mass, and functional loss are poor nutritional status. Further, it is also identified to be a marker for tumor cachexia. In the last 10 years, sarcopenia’s clinical significance for cancer patients has aroused wide attention, such as GC. Yu et al. investigated how sarcopenia plus fibrinogen-albumin ratio (FAR) affected intrahepatic cholangiocarcinoma cases postoperatively (24). The results showed that cases showing both sarcopenia and increased FAR were associated with dismal prognostic outcomes relative to additional patients (24). In addition, sarcopenia plus hepatolithiasis potently predicted the dismal prognostic outcome of intrahepatic cholangiocarcinoma cases receiving curative resection, suggesting the feasibility of sarcopenia plus hepatolithiasis in predicting the prognosis of intrahepatic cholangiocarcinoma cases preoperatively. Another study showed that sarcopenia combined with monocyte-to-lymphocyte ratio was the potent factor predicting OS in breast cancer (BC) cases with lymph node positiveness following mastectomy (25). Sarcopenia together with an increased modified Glasgow prognostic score (mGPS) that contained CRP predicted dismal survival for patients developing locoregional Renal Cell Carcinoma (26). Sarcopenia plus, an increased NLR, predicted the poorer OS of patients with colorectal cancer, stage IV GC, and biliary tract cancer (27–29). PPS is determined according to sarcopenia and LCR and has been suggested as an effective approach to evaluate both the nutritional and inflammatory status of patients with GC (30). As proposed by Xiong et al., PPS was the most effective index for predicting long-term outcomes and recurrence rates for localized GC cases (31). Meanwhile, our study further found that the PPS was a useful factor to predict GCLM prognosis after hepatectomy. Overall, preoperative assessment of inflammation and muscle mass possibly assists in making treatment decision for oncologists, which contributes to identify patients who can gain the best beneficial effects from curative hepatectomy. In addition, it helps us to determine the patients who have a higher disease relapse risk and are considered to receive individualized therapy.

The present work had certain limitations. Firstly, although this study could evaluate the prognostic impact of preoperative PPS in patients with GCLM, selection bias still existed in a retrospective study. Moreover, our unicentric study only enrolled a small sample size and could not establish a validation cohort to further validate the results; therefore, more large prospective studies are warranted. Secondly, we adopted the sarcopenia definition created by Zhuang et al., which deemed sarcopenia criteria for the Chinese GC population (18). The cut off values for the L3-SMI for the diagnosis of sarcopenia were 34.9 and 40.8 cm^2^/m^2^ in men and women. The generalizability of findings in this work was mainly restricted to the Western population since L3-SMI thresholds utilized in this study were specific to geographic regions.

## Conclusion

In conclusion, PPS before surgery represents the simple and effective prognostic factor for patients with GCLM who underwent hepatectomy. The PPS could utilize as a part of precisely predicting GCLM prognosis while improving treatment decision. With the prognostic model, oncologists can determine which cases can probably gain benefits from the aggressive surgery (hepatectomy), thus improving GCLM patient survival.

## Data availability statement

The raw data supporting the conclusions of this article will be made available by the authors, without undue reservation.

## Ethics statement

The studies involving human participants were reviewed and approved by Ethics Committee of National Cancer Center/National Clinical Research Center for Cancer/Cancer Hospital, Chinese Academy of Medical Sciences and Peking Union Medical College. Written informed consent for participation was not required for this study in accordance with the national legislation and the institutional requirements.

## Author contributions

JX and YT conceived of the study and drafted the manuscript. YW, HH, WK, YL, and PJ were responsible for data collection. YW and JX were in charge of statistical analysis. YX, YT, XS, and WL helped to modify the manuscript. All authors contributed to the article and approved the submitted version.

## References

[1] Bray F Ferlay J Soerjomataram I Siegel RL Torre LA Jemal A Global cancer statistics 2018: GLOBOCAN estimates of incidence and mortality worldwide for 36 cancers in 185 countries. *CA Cancer J Clin.* (2018) 68:394–424. 10.3322/caac.21492 30207593

[2] Cheon SH Rha SY Jeung HC Im CK Kim SH Kim HR Survival benefit of combined curative resection of the stomach (D2 resection) and liver in gastric cancer patients with liver metastases. *Ann Oncol.* (2008) 19:1146–53. 10.1093/annonc/mdn026 18304963

[3] Zhang K Chen L. Chinese consensus on the diagnosis and treatment of gastric cancer with liver metastases. *Ther Adv Med Oncol.* (2020) 12:1758835920904803. 10.1177/1758835920904803 32127925 PMC7036491

[4] Muro K Van Cutsem E Narita Y Pentheroudakis G Baba E Li J Pan-Asian adapted ESMO clinical practice guidelines for the management of patients with metastatic gastric cancer: a JSMO-ESMO initiative endorsed by CSCO, KSMO, MOS, SSO and TOS. *Ann Oncol.* (2019) 30:19–33. 10.1093/annonc/mdy502 30475956

[5] Takemura N Saiura A Koga R Arita J Yoshioka R Ono Y Long-term outcomes after surgical resection for gastric cancer liver metastasis: an analysis of 64 macroscopically complete resections. *Langenbecks Arch Surg.* (2012) 397:951–7. 10.1007/s00423-012-0959-z 22615045

[6] Okano K Maeba T Ishimura K Karasawa Y Goda F Wakabayashi H Hepatic resection for metastatic tumors from gastric cancer. *Ann Surg.* (2002) 235:86–91. 10.1097/00000658-200201000-00011 11753046 PMC1422399

[7] Oki E Tokunaga S Emi Y Kusumoto T Yamamoto M Fukuzawa K Surgical treatment of liver metastasis of gastric cancer: a retrospective multicenter cohort study (KSCC1302). *Gastric Cancer.* (2016) 19:968–76. 10.1007/s10120-015-0530-z 26260876

[8] Markar SR Mackenzie H Mikhail S Mughal M Preston SR Maynard ND Surgical resection of hepatic metastases from gastric cancer: outcomes from national series in England. *Gastric Cancer.* (2017) 20:379–86. 10.1007/s10120-016-0604-6 26939792

[9] Kinoshita T Kinoshita T Saiura A Esaki M Sakamoto H Yamanaka T Multicentre analysis of long-term outcome after surgical resection for gastric cancer liver metastases. *Br J Surg.* (2015) 102:102–7. 10.1002/bjs.9684 25389030

[10] Okugawa Y Toiyama Y Yamamoto A Shigemori T Ichikawa T Yin C Lymphocyte-to-C-reactive protein ratio and score are clinically feasible nutrition-inflammation markers of outcome in patients with gastric cancer. *Clin Nutr.* (2020) 39:1209–17. 10.1016/j.clnu.2019.05.009 31155370

[11] Cheng CB Zhang QX Zhuang LP Sun JW. Prognostic value of lymphocyte-to-C-reactive protein ratio in patients with gastric cancer after surgery: a multicentre study. *JPN J Clin Oncol.* (2020) 50:1141–9. 10.1093/jjco/hyaa099 32564084

[12] Cruz-Jentoft AJ Bahat G Bauer J Boirie Y Bruyère O Cederholm T Sarcopenia: revised European consensus on definition and diagnosis. *Age Ageing.* (2019) 48:16–31. 10.1093/ageing/afy169 30312372 PMC6322506

[13] Yang Z Zhou X Ma B Xing Y Jiang X Wang Z Predictive value of preoperative sarcopenia in patients with gastric cancer: a meta-analysis and systematic review. *J Gastrointest Surg.* (2018) 22:1890–902. 10.1007/s11605-018-3856-0 29987739

[14] Kamarajah SK Bundred J Tan BHL. Body composition assessment and sarcopenia in patients with gastric cancer: a systematic review and meta-analysis. *Gastric Cancer.* (2019) 22:10–22. 10.1007/s10120-018-0882-2 30276574

[15] Xiong J Wu Y Hu H Kang W Li Y Jin P Prognostic significance of preoperative sarcopenia in patients with gastric cancer liver metastases receiving hepatectomy. *Front Nutr.* (2022) 9:878791. 10.3389/fnut.2022.878791 35619951 PMC9127608

[16] Xiong J Hu H Kang W Li Y Jin P Shao X Peking prognostic score, based on preoperative sarcopenia status, is a novel prognostic factor in patients with gastric cancer. *Front Nutr.* (2022) 9:910271. 10.3389/fnut.2022.910271 35747263 PMC9210445

[17] Strasberg SM Phillips C. Use and dissemination of the brisbane 2000 nomenclature of liver anatomy and resections. *Ann Surg.* (2013) 257:377–82. 10.1097/SLA.0b013e31825a01f6 22895397

[18] Zhuang CL Huang DD Pang WY Zhou CJ Wang SL Lou N Sarcopenia is an independent predictor of severe postoperative complications and long-term survival after radical gastrectomy for gastric cancer: analysis from a large-scale cohort. *Medicine (Baltimore).* (2016) 95:e3164. 10.1097/md.0000000000003164 27043677 PMC4998538

[19] Wagner AD Unverzagt S Grothe W Grothe W Yong WP Tai BC Chemotherapy for advanced gastric cancer. *Cochrane Database Syst Rev.* (2010) 3:Cd004064. 10.1002/14651858.CD004064.pub3 28850174 PMC6483552

[20] Markar SR Mikhail S Malietzis G Athanasiou T Mariette C Sasako M Influence of surgical resection of hepatic metastases from gastric adenocarcinoma on long-term survival: systematic review and pooled analysis. *Ann Surg.* (2016) 263:1092–101. 10.1097/sla.0000000000001542 26797324

[21] Montagnani F Crivelli F Aprile G Vivaldi C Pecora I De Vivo R Long-term survival after liver metastasectomy in gastric cancer: systematic review and meta-analysis of prognostic factors. *Cancer Treat Rev.* (2018) 69:11–20. 10.1016/j.ctrv.2018.05.010 29860024

[22] Okugawa Y Toiyama Y Yamamoto A Shigemori T Ide S Kitajima T Lymphocyte-C-reactive protein ratio as promising new marker for predicting surgical and oncological outcomes in colorectal cancer. *Ann Surg.* (2020) 272:342–51. 10.1097/sla.0000000000003239 32675548

[23] Nakamura Y Shida D Boku N Yoshida T Tanabe T Takamizawa Y Lymphocyte-to-C-reactive protein ratio is the most sensitive inflammation-based prognostic score in patients with unresectable metastatic colorectal cancer. *Dis Colon Rectum.* (2021) 64:1331–41. 10.1097/dcr.0000000000002059 34623347

[24] Yu H Wang M Wang Y Yang J Deng L Bao W The prognostic value of sarcopenia combined with preoperative fibrinogen-albumin ratio in patients with intrahepatic cholangiocarcinoma after surgery: a multicenter, prospective study. *Cancer Med.* (2021) 10:4768–80. 10.1002/cam4.4035 34105304 PMC8290250

[25] Deng JP Hua X Long ZQ Zhang WW Lin HX He ZY Prognostic value of skeletal muscle index and monocyte-to-lymphocyte ratio for lymph node-positive breast cancer patients after mastectomy. *Ann Transl Med.* (2019) 7:775. 10.21037/atm.2019.11.37 32042791 PMC6990033

[26] Higgins MI Martini DJ Patil DH Nabavizadeh R Steele S Williams M Sarcopenia and modified glasgow prognostic score predict postsurgical outcomes in localized renal cell carcinoma. *Cancer.* (2021) 127:1974–83. 10.1002/cncr.33462 33760232

[27] Shigeto K Kawaguchi T Koya S Hirota K Tanaka T Nagasu S Profiles combining muscle atrophy and neutrophil-to-lymphocyte ratio are associated with prognosis of patients with stage IV gastric cancer. *Nutrients.* (2020) 12:884. 10.3390/nu12061884 32599747 PMC7353220

[28] Lee BM Cho Y Kim JW Jeung HC Lee IJ. Prognostic significance of sarcopenia in advanced biliary tract cancer patients. *Front Oncol.* (2020) 10:1581. 10.3389/fonc.2020.01581 32984018 PMC7492547

[29] Feliciano EMC Kroenke CH Meyerhardt JA Prado CM Bradshaw PT Kwan ML Association of systemic inflammation and sarcopenia with survival in nonmetastatic colorectal cancer: results from the C SCANS study. *JAMA Oncol.* (2017) 3:e172319. 10.1001/jamaoncol.2017.2319 28796857 PMC5824285

[30] Ignacio de Ulíbarri J González-Madroño A de Villar NG González P González B Mancha A CONUT: a tool for controlling nutritional status. First validation in a hospital population. *Nutr Hosp.* (2005) 20: 38–45. 15762418

[31] Kuroda D Sawayama H Kurashige J Iwatsuki M Eto T Tokunaga R Controlling nutritional status (CONUT) score is a prognostic marker for gastric cancer patients after curative resection. *Gastric Cancer.* (2018) 21:204–12. 10.1007/s10120-017-0744-3 28656485

